# Myopia in Bulgarian school children: prevalence, risk factors, and health care coverage

**DOI:** 10.1186/s12886-022-02471-2

**Published:** 2022-06-04

**Authors:** Mila Dragomirova, Albena Antonova, Slavena Stoykova, Gergana Mihova, Denitsa Grigorova

**Affiliations:** 1 Faculty of Physics, Sofia University, 5 James Bourchier Blvd., 1164 Sofia, Bulgaria; 2 Faculty of Mathematics and Informatics, Sofia University, 5 James Bourchier Blvd., 1164 Sofia, Bulgaria; 3 Faculty of Medicine, Sofia University, 1 Koziak Str., 1407 Sofia, Bulgaria; 4 Big Data for Smart Society Institute, Sofia University, 125 Tsarigradsko Shosse, Bl. 2, 1113 Sofia, Bulgaria

**Keywords:** Myopia, Myopia prevalence, Refractive error, Visual acuity, Myopia risk factors, Myopia coverage

## Abstract

**Background:**

The prevalence of myopia has increased in recent years, with changes being dynamic and uneven in different regions. The purpose of this study is to evaluate the prevalence of visual impairment caused by myopia in Bulgarian school children, associated risk factors, and health care coverage.

**Methods:**

A cross-sectional study among 1401 children (mean age 10.38, standard deviation 2.70) is conducted in three locations in Bulgaria from 2016 to 2020. Refractive error is measured with an auto-refractor in the absence of cycloplegia, the visual acuity is assessed without refractive error correction. A paper-based preliminary questionnaire is used to collect data on previous eye examinations, prescribed optical vision correction, regularity of wearing corrective glasses and risk factors.

**Results:**

Children with myopic objective refraction ≤ -0.75 D and decimal visual acuity ≤ 0.8 on at least one of the eyes are 236 out of 1401 or 16.85%. The prevalence of myopia varies depending on age, geographical location, and school profile. The rate of myopic children in age group 6–10 is 14.2% compared to 19.9% in age group 11–15. The prevalence of myopic children in the urban populations is 31.4% (capital) and 19.9% (medium-sized town) respectively, and only 8.4% in the rural population. Our results show 53% increase of the prevalence of myopia in the age group 11–15 compared to a 2009 report. The analysis of data associated with health care coverage factors of all myopic pupils shows that 71.6% had a previous eye examination, 43.2% have prescription for corrective glasses, 27.5% wear their glasses regularly. Risk factors for higher odds of myopia are gender (female), age (adolescence), and parents with impaired vision. Residence in a small town and daily sport activities correspond to lower odds for myopia. The screen time (time in front of the screen calculated in hours per day) is self-reported and is not associated with increased odds of myopia when accounted for the other risk factors.

**Conclusions:**

The prevalence of myopia in this study is higher compared to previous studies in Bulgaria. Additional studies would be helpful in planning adequate prevention and vision care.

**Supplementary Information:**

The online version contains supplementary material available at 10.1186/s12886-022-02471-2.

## Introduction

The occurrence of myopia has gained importance in epidemiological studies due to its increasing prevalence, especially among young people and children [1–3]. Myopia is the most common cause of distant vision impairment [1, 3]. Uncorrected myopia in children can affect school performance and lead to lower quality of life, impacting the individual and the community [4, 5]. High-level myopia significantly increases the risk of developing several ocular complications such as retinal detachment, glaucoma, cataract, optic disk changes and maculopathy at a later age [3, 4]. Age of myopia onset and duration of myopia progression is the most significant prognosticator of high myopia later in life [4, 6, 7].

During the last years, many studies have found an increased myopia prevalence in young people and children, rising dramatically to the level of 60–80% in East Asian countries and ∼25–40% in Western countries [1, 2, 8, 9]. In 2000 the number of myopic people is about 1.4 billion, estimating its rise to 4.8 billion by 2050 [1]. The accelerating myopia prevalence rates are expected to lead to considerable public health challenges [1, 7, 8].

All this confirms that proper health care coverage is essential to avoid higher rates of vision impairment caused by myopia. Thus, all people with myopia should have access to appropriate, accurate refractive correction [5, 7]. Furthermore, identifying and assessing the risk factors for myopia can improve the prevention actions. For example, among the main factors associated with a higher risk of myopia are time spent near work, less time outdoors, higher educational level and parental history [10–12]. Several studies confirm the positive effects of the time spent outdoors and other behavioural changes on myopia onset and progression in children [10–12]. Spending more time outdoors can prevent the onset of myopia, lower the effect of near work and the heredity effect from parental myopia, possibly slowing the myopia progression rate [10, 11].

Although parental myopia is usually considered to be a risk factor for myopia in children [12], according to new papers, parental myopia is not necessarily a genetic factor [13]. Recent research provides evidence that genetic and environmental risk factors have an equally large impact on myopia [14]. The possibility that myopic parents might create myopiagenic environments should be taken into account and there should be greater emphasis on lifestyle adjustments in myopia prevention.

The rate of myopia prevalence varies across populations of different regions and ethnicities. The population‐based studies report a higher prevalence of myopia among children in urban areas and within East-Asian countries [1]. Other studies observe specific deviations in myopia prevalence depending on age, sex, occupation, environment, socio-economic factors [1, 3, 12]. However, directly comparing the outcomes of different studies is not appropriate, due to differing methodologies and the lack of statistically reliable epidemiological data for myopia prevalence for many countries and regions [3, 7]. The lack of standardized methodologies, criteria set and available data, hinder further the opportunity to propose relevant policy measures and health care management plans to control myopia prevalence and risk factors across countries and regions [7].

The purpose of the present study is to evaluate the prevalence of visual impairment caused by myopia in Bulgarian school children, associated risk factors and health care coverage. It aims to provide additional evidence and up-to-date analysis on myopia prevalence and risk factors for Bulgarian school children. Conforming to the literature, epidemiological studies and the provision of up-to-date databases are essential for the health system to plan for the care of impaired vision and to monitor the prevalence, coverage, and risk factors of myopia.

## Methods

### Study participants

The cross-sectional study was conducted from November 2016 to January 2020, covering four schools in three locations in Bulgaria. All four schools are public, three of them deliver both primary and secondary education, and one is a secondary school specialised in sport. In Bulgaria the education system is organized in the following levels: primary (usually students aged 6—10 years old), secondary (students aged 10—15 years old) and high school (students aged 15—19 years old). Using the system described above, primary and secondary school children are included in the current study. Three of the schools are in urban regions: two are in Sofia (the capital), one is in Veliko Tarnovo (medium-sized town) and the fourth school is in a rural area – in Devnya (small-sized town). All pupils from the selected schools were invited to participate voluntarily, and 1401 of them took part in the study. According to the National Statistical Institute, about 3/4 of the Bulgarian population live in urban regions and 1/4 live in rural areas [15]. Of the 1401 children in the selected sample, 1066 live in urban areas and 335 in a rural area, which is suitable for the sample representativeness. In addition, all four schools are public, so they are open to children from all socio-economic backgrounds and diverse demographic groups are represented. The design of the study is observational, and no intervention procedures are made.

The next short overview of on-the-field work explains the three stages of examinations held from 2016 to 2020.

The first stage was completed in November 2016 in *Vasil Levski School* in Devnya: a small town with 9 500 residents located in a rural area in the North-East part of the country. All pupils present at the time of schooling took part in the survey, in total 335 participants, 168 boys and 167 girls. The mean age of the children in the study is 10.60 years, and the standard deviation is 3.52.

The second stage took part in *Bacho Kiro School*, in Veliko Tarnovo, a regional centre with approximately 70 000 residents located in the North-Central region. The examinations were performed during the two visits of the team (in March and April 2019) due to the large number of pupils: in total 748 participants, 379 boys and 369 girls. The mean age is 9.73, and the standard deviation is 2.11.

The third stage was performed in Sofia, in two different schools. Sofia is the capital of Bulgaria, with an official population of 1 240 000 residents and an unofficial estimation of 2 million residents. The study in the *Sports school Gen. Vladimir Stoychev* was performed at the end of October 2017. The Sports school is a secondary school specialized for children with sport talents, providing everyday sport and training activities. In total, 181 participants from the school took part in the study, 121 boys and 60 girls. The mean age is 13.29, and the standard deviation is 1.11. Last, in January 2020 the last part the study is in *Vasil Aprilov School*, located at Gorublyane district in the outskirts of Sofia. A total of 137 school children participated, 75 boys and 62 girls. The mean age is 9.46, and the standard deviation is 2.05.

### Experimental procedure

The presence of myopia is determined using the criteria: Spherical equivalent ≤ -0.75 D and decimal VA ≤ 0.8 (corresponding to logMAR acuity ≥ 0.1) in at least one of the eyes. Additionally, calculations of the count and simple proportions were made using three different criteria for myopia: (SE ≤ -0.5 D); (SE ≤ -0.5 D and decimal VA ≤ 0.8); (SE ≤ -0.75 D and decimal VA ≤ 0.8). First, the standard criterion of -0.5 E was used. Then, since the study was performed without cycloplegia, a criterion for visual acuity of decimal 0.8 was added to reduce the effect of overestimation of myopia. The above calculations have been made to address the use of non-cycloplegic refraction, which is known to overestimate myopia in children [16, 17]. Uncorrected VA is used as additional criterion to noncycloplegic assessments to better detect myopia [18]. The spherical equivalent refraction (SER) is calculated as sphere plus half of the cylindrical error. The sphero-cylindrical refractive error is measured without optical correction and any cycloplegic drugs, using an auto-refractor with an auto fogging system for relaxation of accommodating eye (Nidek AR-310A) [19]. All manufacturer’s instructions for use of the equipment are followed to avoid inaccuracies in the results obtained. Before the measurements with the auto-refractor, each participant received an explanation of the procedure and instructions.

Visual acuity (VA) is measured monocularly without refractive correction and any cycloplegic drugs, using a logMAR type chart. Tumbling E optotypes were used for the measurement, considering that cyrilic letters are more familiar for young children in Bulgaria. A standard visual screen (Medizs, Korea), at 5 m distance, held the acuity charts. Luminance values are within the recommendations for standardizing the measurement of VA. Decimal VA is measured by-line. Decimal scores are converted to logMAR using the formula logMAR = -log(decimal acuity). All subjects are asked to identify the optotype one by one in each line, starting from the upper left letter. They are instructed to read slowly and guess the letters when they are unsure. A termination rule of 3 or more mistakes on a completed line is used. The clinicians performing the measurement are pre-trained to follow the study methodology. The survey was conducted in schools during school hours, in order to include all students present that day. It would not have been possible to use cycloplegic refraction to define myopia, as in Bulgaria the use of cycloplegic drugs is allowed only in clinics and ophthalmology offices.

A paper-based questionnaire is used before the examination to identify the health care coverage and the risk factors. The questionnaire is distributed in advance and must be filled by the parents of the younger children, or by the children (over the age of 10). The questionnaire aimed to obtain additional information about the risk factors, clarifying if the parents wear glasses, the screen time (how many hours per day the child spent in front of a screen) and sports activities per week. The health care coverage information is defined as previous child’s eyes examinations, prescribed optical correction of vision and the proportion of children wearing permanently prescribed glasses.

## Statistical analysis

The statistical analyses are performed using RStudio [20]. For some variables missing values are observed, due to lack of responses to some of the questions. In all analyses, a pairwise deletion is used to cope with the missing data except for one variable for which we did imputation (explained in the last paragraph of Limitations of study section).

Concerning responses to the risk factors, obtained in the paper-based questionnaire, the following groupings are made. Sport activities are reported in days per week. For the analysis, the children are divided into two groups: children playing sport every day versus not playing sport every day (rarely or never), making the variable binary.

The screen time is reported in hours per day, spent in front of the screen. The screen could be any type of monitor or device such as TV, smartphone, game console, computer, laptop, tablet or other. Based on the responses, two groups are formed—less than 4 h versus more or equal to 4 h of screen time per day (the threshold of 4 h is chosen based on the answers thinking that 4 h screen time or more is above than the norm).

First, statistical analysis is performed to establish if the proportions of myopia are the same among the different groups according to the variables of the risk factors (school, age, etc.). The logistic regression model is fitted with the risk factors as predictors to see which ones are significant given the others. Backward elimination is used to remove the insignificant variables. A test is performed to check if the proportions of the levels of different grouping variables are equal only among the children with myopia using chi square test for given probabilities. The significance level for all statistical analyses is set to 5%.

## Results

The counts and sample proportions of children with myopia for the three chosen myopia criteria, distributed by different schools, are presented in Table 1.

**Table 1:** Counts and sample proportions of children with myopia using three different myopia criteria

Grouping characteristic	Count (Sample proportions)			
School	Rural (Small tawn)	Urban (Capital)	Urban—Sport (Capital)	Urban (medium sized town)
SE ≤ -0.5 D	173 (51.64%)	74 (54.02%)	75 (41.67%)	418 (56.03%)
SE ≤ -0.5 D and decimal VA ≤ 0.8	33 (9.85%)	47 (34.31%)	17 (9.39%)	169 (22.59%)
SE ≤ -0.75 D and decimal VA ≤ 0.8	28 (8.36%)	43 (31.38%)	16 (8.84%)	149 (19.91%)

The outcomes of the present study indicate that 236 (16.85%) from all 1401 children have a myopic visual impairment (SE ≤ -0.75 and decimal VA ≤ 0.8) in at least one of the eyes. A more detailed analysis of the sample proportions of the children with myopia is provided in Table 2. The percentage of the myopic children within the sample varies depending on age, geographical location, and school profile. Among all 759 children aged ≤ 10 years (at the first school level), 108 (14.22%) are myopic. Among all 642 children above 10 years old (at the secondary school level), the myopic children are 128 (19.9%). Considering gender, boys with myopia are 103 (13.86%) of all 743 boys and girls with myopia are 133 (20.21%) of all 658 girls. The average values of myopic refraction in the two age groups are: for the right eye -2.08 D and -1.62 D for the Secondary school and Primary school age groups respectively, while for the left eye the averages are -1.94 D and -1.39 D for the same age groups. The average values of refraction for the group of emmetropes (SE > -0.75D/ < 0.75D), for the whole sample are: for the right eye (RE) -0.24 D and -0.15 D for the age groups 11–15 and 6–10 respectively, while for the left eye (LE) the averages are -0.09 D and -0.02 D. The refraction in children with hyperopia from the whole sample shows the following averages: RE 1.1 D and 1.19 D for the age groups 11–15 and 6–10 respectively, while for LE averages are 1.23 D and 1.38 D. Additionally, average refractive values were calculated for the subgroup that showed the lowest prevalence of myopia for ages 11–15: the school specializing in sports. The average values are: emmetropes RE -0.1 D and LE -0.06 D, respectively for hyperopes RE 1.69 D and LE 1.64 D.

**Table 2:** Counts and sample proportions of children with myopia for the different grouping variables which we call risk factors:

Grouping characteristic	Count (Sample proportions)			
School	Rural(Small tawn)	Urban (Capital)	Sport(Capital)	Urban (medium sized town)
	28 (8.36%)	43 (31.38%)	16 (8.84%)	149 (19.91%)
Age	6 - 10	11 - 15		
	128 (19.93%)	108 (14.22%)		
Gender	Male	Female		
	103 (13.86%)	133 (20.21%)		
Sport	Every day	Not every day		
	48 (11.74%)	186 (19.60%)		
Screen time	Less than 4 hours	More or equal to 4 hours		
	179 (17.92%)	51 (14.87%)		
Previous examination	Yes	No		
	67 (9.81%)	169 (23.93%)		
Parents wear glasses	None of the parents	At least one parent		
	113 (13.47%)	114 (23.46%)		

Given that at least one parent wears glasses 114 children are myopic (23.46%). While the number of the myopic children in the group where none of the parents wear glasses are 113 in total or only 13.47% (Table 2).

The next statistical analysis is performed to establish if the proportions of myopia are equal among the different groups according to the same grouping variables (school, age, etc.). The results are presented in Table 3.

**Table 3:** Chi square test for comparing equal proportions of myopia for the different risk factors

Grouping characteristic	Chi^2 test statistic	Degrees of freedom	*P*-value
School	51.237	3	< 0.0001
Age group (above and below 10)	7.689	1	0.0056
Gender	9.597	1	0.0019
Sport (every day versus not every day)	11.847	1	0.0006 (43 missing observations)
Time in front of the screen (less than 4 h versus more or equal to 4 h)	1.4638	1	0.2263 (59 missing observations)
Previous examinations	48.134	1	< 0.0001 (12 missing observations)
Parents wear glasses (at least one of the parents to wear glasses versus none of them)	20.928	1	< 0.0001 (76 missing observations)

**Fig. 1:**
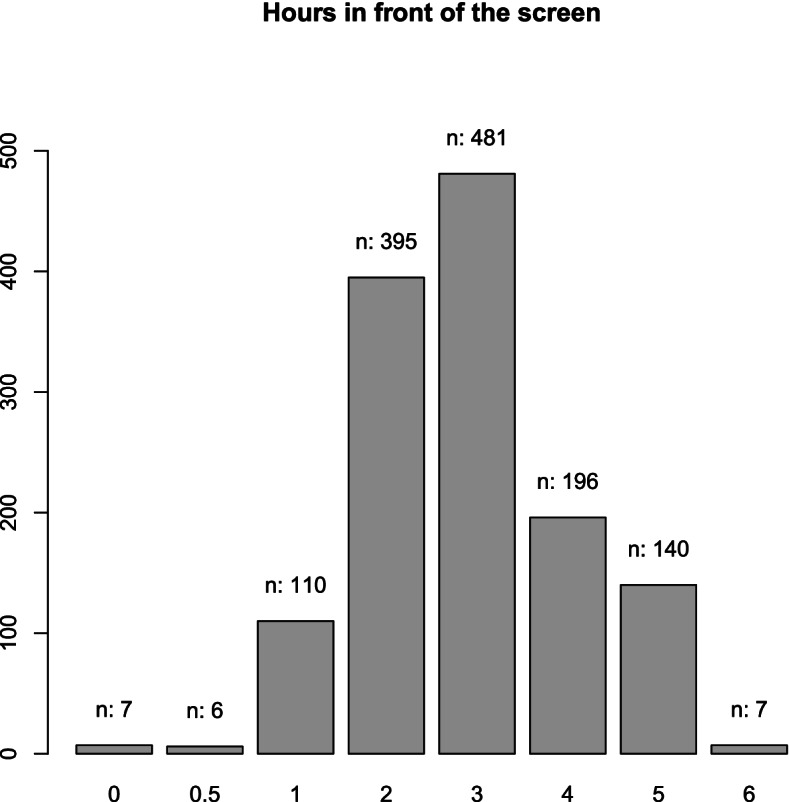
Barplot of the variable time in front of the screen (in hours) per day

Statistically different (at 5% significance level) are the proportions for the following risk factors: school, age, gender, sport (every day versus not every day), previous examinations, whether parents wear glasses (at least one of the parents versus none of the parents). Statistically non-significantly different are the proportions of myopic children in the two groups depending on the time spent in front of the screen (less than 4 h versus more or equal to 4 h per day). Figure 1 shows the original distribution of the variable screen time.

The health care coverage analysis for the children with myopia and the proportions of the levels of the different grouping variables are presented in Table 4. Among all myopic pupils, 169 (71.61%) have previous eye examination, 102 (43.22%) have prescribed corrective glasses.

**Table 4:** Proportions of the levels of different grouping variables among myopic children

Grouping characteristic	Sample proportions			
Examination before (yes or no)	No		Yes	
	0.284		0.716	
	95% CI for No: (0.228, 0.347)			
Have glasses (yes or no)	No		Yes	
	0.568		0.432	
	95% CI for No: (0.502, 0.632)			
Wear glasses (yes, no, sometimes)	No	Sometimes		Yes
	0.648	0.076		0.275

The next step is to test whether the proportions of the levels of the different grouping variables only for the children with myopia are equal using chi square test for given probabilities. The results are summarized in Table 5. From the table we see that all of the tests have *p*-values less than 5%, which means that the proportions of the levels are not equal for each considered grouping variable.

**Table 5:** Chi-square test for equal probabilities of the levels of the grouping variables among the myopic children

Grouping characteristic	Chi^2 test statistic	Degrees of freedom	*P*-value
Examination before (yes or no)	44.085	1	< 0.0001
Children have glasses (yes or no)	4.339	1	0.0373
Children wear glasses (yes, no, sometimes)	119.4	2	< 0.0001

A logistic regression model for having myopia or not is fitted with predictors the risk factors variables from Table 2 in order to identify only the significant variables. Backward elimination with 5% significance level is used to remove the insignificant variables. The estimates of the odds ratios and their 95% confidence intervals from the final model are given in Table 6.

**Table 6:** Estimates and 95% confidence intervals of the odds ratios in the logistic model with only significant risk factor variables

Variable	Estimate	Lower Bound 95% CI	Upper Bound 95% CI
Town: Sofia-Gorubliane	4.643	2.712	8.064
Town: Sofia-Sport	0.580	0.278	1.160
Town: Veliko Tarnovo	2.337	1.524	3.696
Gender (Female)	1.595	1.184	2.155
Age group: below 10	0.550	0.404	0.750
Parents wear glasses: at least one of them	1.677	1.239	2.271

## Discussion

Among the main conclusions from the statistical analyses is the finding that as much as 28.39%, 95% CI: (22.82%, 34.67%) of all myopic children did not have previous examinations, which demonstrates a considerable lack in the health care coverage. Even more alarming is the discovery that as much as 56.78%, 95% CI: (50.19%, 63.15%) of all myopic children don’t have glasses although they need them. Only 65 myopic children (out of 236) wear glasses all the time (point estimate: 27.54%; 95% confidence interval: (22.04%, 33.79%)) and 83 wear glasses all the time or sometimes (point estimate: 35.17%; 95% confidence interval: (29.16%, 41.67%)). The results of the logistic regression (Table 5) highlights that girls (compared to boys), children that have at least one parent wearing glasses (compared to children whose parents don’t wear glasses), children from urban schools Gorubliane (Sofia) and Veliko Tarnovo (compared to rural, those from Devnya) have increased odds of myopia. Concerning age, children below 10 years have lower risk of myopia compared to the children above 10. The children from the Sport school (Sofia) have lower risk of myopia compared to the children from Devnya but the odds ratio is not statistically different from 1.

Considering the outcomes of the presented cross-sectional study, the obtained results are comparable to other published epidemiological studies in Europe, reporting myopia prevalence as follows: Poland (7 years 4.0%, 12 years 14.4%), Ireland (6–7 years 3.3%, 12–13 years 19.9%), the UK Northern Ireland Childhood Errors of Refraction (NICER) study (6–7 years 2.8%, 12–13 years 17.7%), Aston Eye Study (AES) (6–7 years 5.7%, 12–13 years 18.6%) [21–23]. The results are slightly lower in Australia (6 years 1.6%, 12 years 12.8%) [24]. A direct comparison of the prevalence of myopia should not be done, due to the different methods and criteria used to measure it: with or without cycloplegia, auto-refractometer, retinoscopy, subjective refraction, and a cut-off value ranging from -0.25 to -1.0 D in different studies [8].

Regarding Bulgarian children, a small number of epidemiological studies on myopia are available. To the best of our knowledge, there is only one paper [23] that reports the prevalence of myopia in children in the same age groups in Bulgaria [25]. Plainis et al. (2009) measured non-cycloplegic refraction and set the cut-off value for myopia to -0.75D. A VA criterion (0.8 Decimal) was also used, in order to eliminate most of the pseudo-myopes. In our study we used the same methodology which allows for a more accurate comparison of possible changes in the prevalence of myopia. Plainis et al. (2009) find that the percentage of myopic children is 14.1% at primary school level and 13.0% at secondary school level for children in Stara Zagora (Bulgaria). Among the myopic pupils, only 35.8% of Bulgarian children are reported to wear corrective spectacles [25]. The data on 310 Bulgarian children in the study of Plainis et al. are collected in Stara Zagora, a Bulgarian town similar to Veliko Tarnovo in terms of population and socio-economic development. Our results show a higher prevalence of myopia among children of the older age group and also a similar percentage of children with optical correction of their myopia. Our results show 1% increase of the prevalence of myopia in primary school and 53% increase in secondary school compared to the results of Plainis et al. (2009). However, the results of Plainis should be interpreted with caution due to the atypical distribution of myopia in the two age groups: the finding that the prevalence of myopia is lower in secondary than primary level is quite unusual. A possible explanation could be that the study was accidentally conducted at a time when the prevalence of myopia was beginning to increase specifically in the generation of children who were in the younger age group at the time of the study. Unfortunately, the lack of other studies within this population during that period makes drawing any conclusions difficult. Additional research would be useful to provide more evidence for the potential increase in the prevalence of myopia.

The study by Slaveykov and Trifonova (2020) [26] publishes data about examination of 596 children aged 3–6 years in Kazanlak, a town with a population of 44 760 residents. Myopia is defined as SE ≤  − 0.50 D [24]. The children underwent non-mydriatic refraction screening using the Plus-optix S12c Mobile camera. The study reports a myopia rate of 6.8% to 9.3% for different age groups, highlighting that 33% of the children with myopia have never visited ophthalmologist.

Another trend, concerning the age distribution of myopia prevalence is also observed in our study. This confirms the finding that age is the most critical parameter for epidemiological analysis of myopia worldwide, and the prevalence rate of myopia increases significantly with age [8, 23]. Therefore, preventing early onset of myopia requires a collaborative effort among professionals, health care institutions, schools, parents, and the entire society.

In the first place, it should be noted the parents’ role for the prevention and early treatment of myopia. Usually, the impaired vision of the parents is associated with higher odds of myopia [12, 13] (of which our data show evidence), and this can be linked not only to genetic inheritance but also to the inherited family lifestyle and habits, such as time spent near work, daily sport trainings and outdoor activities. Parental attitudes can affect the health coverage of the child’s myopia: paying attention to diagnose myopia on time, providing glasses for optical correction of the child’s vision and motivating regular wearing of the glasses.

In our study, surprisingly, the screen time is not associated with different odds of myopia, given that all other risk factors considered are included in the logistic model (*p*-value = 0.124) and when this is the only risk factor considered (*p*-value = 0.226, Table 3). One of the explanations could be that the data are not reported correctly, relying on self-reports of the children or parents, biasing intentionally or not the time spent in front of a smartphone or a computer. In the literature, when using an objective method to measure the time of smartphone use (as the amount of data used), the correlation with myopia is clearly observed. According to MacCrann et al., myopic students use almost double the amount of smartphone data per day compared to non‐myopic, while the smartphone time use does not significantly differ [27]. More interestingly, the screen time and its role for myopia onset should be more carefully examined following the COVID-19 pandemic measures in 2020 and 2021 and the imposed online learning alternatives. Further studies need to investigate the effects of COVID-19 on the epidemiology of myopia, as it changed profoundly the daily routine of the school-age children, reducing the time for sport and outdoor activities.

### Limitations of this study

In the study design, it was chosen to measure refractive error using an autorefractor in the absence of cycloplegia. This decision was made based on the following consideration: regulations in Bulgaria do not allow the use of cycloplegic drugs outside clinics and ophthalmology offices. In order to obtain a larger sample, the measurements were carried out in schools. The advantages of the chosen methodology are that it does not cause discomfort to children, nor any significant disruption of the learning process. It also makes obtaining parental consent easier. To avoid overestimation of the prevalence of myopia, the condition uncorrected visual acuity < 0.8 has been added to the definition.

The present study has terminated earlier than planned due to the outbreak of Covid 19 pandemic. During the 2020 lockdowns, serious changes are imposed to the daily life of all school children: longer time in front of screens, limited time outside and almost lack of outdoor sports activities. It would be interesting to conduct a similar study after the pandemic ends and compare the new data for myopia prevalence.

To some questions from the questionnaire the children/parents did not give an answer. We substituted answers “No” to the missing values of the variable if the children wear glasses. We examined more closely the children with missing values for this variable (141 in total) and 134 of them don’t have glasses (67 of them did not have even an examination before and 67 had an examination). Only 7 children have an examination before and have prescribed glasses. We believe that such manipulation of the data is justifiable. Regarding the missing values in the other variables, we only did pairwise deletion for all analyses.

The current study focuses specifically on children in primary and secondary school (levels as defined in the Bulgarian school system). Future research could examine myopia in high school students. The senior years of high school are of particular interest, since onset and progression of myopia have generally slowed by that age [1, 7]. Such data could reveal what prevalence is likely to spread through the population as the present cohort ages and would show expected future trends in Bulgaria.

The present study focuses on the presence of myopia and does not aim to analyze the levels of myopia in students of the included age groups. In addition, it would be interesting to collect and analyze data on hyperopia and astigmatism in these populations.

## Supplementary Information

**Additional file 1.** 

## Data Availability

The datasets used and/or analysed during the current study are available from the corresponding author on reasonable request.
